# Early Evolution of Sepsis-Associated Coagulopathy and Its Association with Therapeutic Exposures: A Prospective Longitudinal Cohort Study

**DOI:** 10.3390/jcm15166285

**Published:** 2026-08-13

**Authors:** Gianni Turcato, Lucia Filippi, Arian Zaboli, Fabrizio Lucente, Michael Maggi, Paolo Ferretto, Daniela Milazzo, Alberto Caregnato, Alice Bresolin, Alessandra Eugenia Bionda, Christian J. Wiedermann, Lorenzo Ghiadoni

**Affiliations:** 1 Intermediate Care Unit, Department of Internal Medicine, Hospital Alto Vicentino (AULSS-7), 36014 Santorso, Italy; gianni.turcato@yahoo.it (G.T.); fabrizio.lucente@aulss7.veneto.it (F.L.); michael.maggi@aulss7.veneto.it (M.M.); paolo.ferretto@aulss7.veneto.it (P.F.); daniela.milazzo@aulss7.veneto.it (D.M.); alberto.caregnato@aulss7.veneto.it (A.C.); alice.bresolin@aulss7.veneto.it (A.B.); 2 Department of Health Sciences, UniCamillus–Saint Camillus International University of Health Sciences, 00131 Rome, Italy; 3 Health Professions Management, South Tyrolean Health Authority (SABES-ASDAA), 39100 Bolzano, Italy; zaboliarian@gmail.com; 4 Emergency Department, Nuovo Santa Chiara Hospital, Azienda Ospedaliero-Universitaria Pisana, 56124 Pisa, Italy; a.e.bionda@gmail.com; 5 Institute of General Medicine and Public Health, Claudiana, 39100 Bolzano, Italy; christian.wiedermann@am-mg.claudiana.bz.it; 6 Department of Clinical and Experimental Medicine, University of Pisa, 56124 Pisa, Italy; lorenzo.ghiadoni@unipi.it

**Keywords:** sepsis-induced coagulopathy, disseminated intravascular coagulation, anticoagulation

## Abstract

**Background:** Coagulopathy in sepsis represents a dynamic continuum ranging from sepsis-induced coagulopathy (SIC) to disseminated intravascular coagulation (DIC), but its early evolution and clinical determinants remain poorly defined. **Objectives:** To assess the temporal dynamics of SIC and DIC and examine factors associated with their evolution during the early phases of sepsis. **Methods:** A prospective longitudinal observational study was conducted on 299 patients with sepsis admitted to the Intermediate Care Unit (IMCU). Patients were evaluated at admission and subsequently at 24, 48, 72, and 96 h, for a total of 1.447 observations. Clinical, laboratory, and hemodynamic data were collected at each time point, and SIC and DIC scores were calculated. The evolution of coagulopathy and its association with clinical and therapeutic variables were analyzed using appropriately adjusted generalized estimating equation (GEE) longitudinal models. **Results:** The prevalence of SIC increased from 36.8% at baseline to 47.5% at 24 h, and then declined to 20.8% at 96 h. DIC prevalence decreased from 21.7% to 9.7%. Coagulopathy at the previous time point was the main determinant of subsequent coagulopathy (SIC: OR 31.17; DIC: OR 67.17; *p* < 0.001). Incidence was highest during the early phases (SIC: 12.8% to 2.2%; DIC: 4.7% to 1.1%), whereas persistence decreased over time (SIC: 34.7% to 18.6%; DIC: 18.5% to 8.6%). Higher Sequential Organ Failure Assesment (SOFA) scores were associated with increased risk. Therapeutic anticoagulation was inversely associated with subsequent coagulopathy, including overt DIC positivity among patients without overt DIC at baseline (OR 0.059; 95% CI 0.012–0.290; *p* < 0.001). Higher cumulative fluid balance was associated with overt DIC in exploratory predicted-probability analyses, independent of vasopressor use, although this gradient did not reach statistical significance in adjusted models and is hypothesis-generating. Diuretic therapy was associated with an increased risk of SIC (OR 2.20; *p* = 0.008). **Conclusions:** Coagulopathy in sepsis occurs early and is strongly dependent on its initial trajectory. SIC and DIC represent stages of a continuum, with onset occurring predominantly within the first 24–48 h. The inverse association with therapeutic anticoagulation is hypothesis-generating and should not be interpreted as a treatment effect.

## 1. Introduction

Sepsis is a complex clinical syndrome characterized by a dysregulated host response to infection, resulting in cellular and tissue injury mediated by systemic inflammation [1,2]. Among the pathophysiological mechanisms underlying sepsis, the activation of coagulation represents a key component, closely interconnected with inflammation and endothelial dysfunction, leading to a state of immunothrombosis that is initially adaptive but may ultimately contribute to microvascular injury and impaired tissue perfusion [3,4,5,6].

Over time, sepsis-associated coagulopathy has been conceptualized as a dynamic spectrum of alterations, ranging from subclinical laboratory abnormalities to sepsis-induced coagulopathy (SIC) and overt disseminated intravascular coagulation (DIC). SIC, introduced by the International Society on Thrombosis and Haemostasis (ISTH) as a tool for the early identification of coagulation abnormalities, represents an initial and potentially reversible stage of the process, whereas DIC constitutes a more advanced stage associated with greater clinical severity and poorer prognosis [7,8]. SIC almost invariably antedates overt DIC, and approximately one third of patients with SIC progress to overt DIC within the first days [7,8]. Its reported prevalence varies markedly across regions, from 40 to 60% in Asian cohorts to approximately 22–24% in European Sepsis-3 cohorts, in which SIC is typically present at sepsis diagnosis or develops within the following four days and is independently associated with mortality [2]. However, despite this classification, the temporal evolution of coagulopathy in septic patients and its patterns of progression or recovery remain incompletely characterized, and in particular the rate of spontaneous resolution during the first days of treatment is insufficiently defined—a gap proposed as one reason why interventional trials targeting septic coagulopathy have repeatedly failed [9,10].

The presence of coagulopathy is known to be associated with worse clinical outcomes. Serial rather than single measurements appear to carry the greater prognostic information: dynamic coagulation patterns, including early fibrinolytic shutdown, have shown stronger associations with mortality than baseline values, yet their fine temporal kinetics remain incompletely defined [5], and ascending biomarker trajectories independently predict mortality in SIC [6]. Nevertheless, its course during the early phases of sepsis and the impact of initial therapeutic strategies remain poorly defined. In particular, interventions aimed at restoring perfusion and hemodynamic stability may indirectly influence coagulation dysfunction, also through modulation of endothelial injury, although this aspect has not yet been adequately clarified. This question is clinically relevant because anticoagulant strategies are thought to be most effective before the transition to overt DIC, when the coagulation trajectory may still be modifiable [7,8]. In this context, the longitudinal assessment of coagulopathy during the early stages of hospitalization, in relation to the therapies administered, may provide relevant insights into its evolution and help identify potential therapeutic targets.

The aim of this study was to characterize the temporal dynamics of SIC and overt DIC in a cohort of patients with sepsis by evaluating their prevalence, incidence, persistence, and resolution during the early phases of the disease. Secondly, we examined the longitudinal determinants of coagulopathy, including clinical, hemodynamic, and therapeutic variables, in order to identify factors associated with its evolution over time.

## 2. Materials and Methods

### 2.1. Study Design

This was a prospective longitudinal observational study conducted in the IMCU of the Alto Vicentino Hospital in Santorso, Italy, between January 2024 and June 2025, and was conducted and reported in accordance with the STROBE recommendations for observational studies. The cohort was drawn from the ongoing prospective sepsis registry of the Alto Vicentino Intermediate Care Unit (IMCU) that has previously served to address distinct research questions [4,9,10]; the present analysis is methodologically independent and focuses specifically on the longitudinal dynamics, transitions, and determinants of SIC and overt DIC over the first 96 h. All consecutive adult patients admitted to the IMCU from the Emergency Department (ED) with a diagnosis of sepsis were included. Sepsis was defined according to the Sepsis-3 criteria as suspected or documented infection associated with an increase of ≥2 points in the Sequential Organ Failure Assessment (SOFA) score [2].

Exclusion criteria were age < 18 years, pregnancy or suspected pregnancy, transfer from wards other than the ED, sepsis occurring in the postoperative or post-traumatic setting within the previous month, ED stay longer than 6 h before admission, and administration of more than 1.000 mL of crystalloids within the three hours preceding admission. Patients with terminal illness and a life expectancy of less than 3 months were also excluded, as were subjects requiring immediate intensive organ support already initiated in the ED or not initiated because of treatment limitations or ineligibility for intensive care.

According to the local admission protocol, patients were admitted to the IMCU when they required intensive monitoring and non-invasive organ support, including invasive hemodynamic monitoring, high-flow oxygen therapy or non-invasive ventilation, and vasoactive support, without an immediate indication for advanced invasive organ replacement. Patients requiring invasive mechanical ventilation, or advanced simultaneous support of multiple organs, were admitted directly to the ICU and were therefore not eligible for the study. Subsequent clinical deterioration requiring these interventions prompted transfer from the IMCU to the ICU.

The study was approved by the local Ethics Committee (protocol no. 406; approval date 3 October 2023) and conducted in accordance with the Declaration of Helsinki. Written informed consent was obtained from all patients or their legal representatives.

### 2.2. Study Protocol

#### 2.2.1. Admission

All patients with suspected infection evaluated in the ED underwent the laboratory investigations required for calculation of the Sequential Organ Failure Assessment (SOFA) score, in order to allow early identification of sepsis. At the time of IMCU admission, the main demographic and clinical data (age, sex, weight, height, and BMI) as well as major comorbidities were systematically collected and used to calculate the Charlson Comorbidity Index (CCI). Simultaneously, vital signs required for the calculation of the National Early Warning Score (NEWS) were recorded.

Upon ward admission, an arterial line was inserted for invasive monitoring and serial blood sampling, together with a urinary catheter for accurate urine output monitoring.

At enrollment, arterial blood gas analysis including lactate measurement was performed, together with blood sampling for complete blood count, electrolytes, renal and liver function parameters (including ALT, AST, total bilirubin, and serum albumin), coagulation profile (PT-INR, aPTT, fibrinogen, and D-dimer), blood glucose, and inflammatory markers (C-reactive protein and procalcitonin).

Based on the data collected at admission, APACHE II and SOFA severity scores were calculated using only parameters obtained before initiation of any therapeutic intervention. SIC and ISTH overt-DIC scores were also calculated.

At enrollment, anticoagulant therapy was recorded according to the agent, dose, and administration schedule. Prophylactic-dose anticoagulation consisted of [drug and exact dose/frequency], with dose adjustment for [renal function/body weight, if applicable]. Therapeutic-dose anticoagulation consisted of [drug and exact dose/frequency or weight-based regimen], prescribed for an established clinical indication. Classification was based on the dose actually administered at enrollment.

#### 2.2.2. Reassessment

All patients enrolled in the study underwent reassessment at 24 h intervals, for a total of four reassessments in addition to enrollment. At each reassessment, the same blood tests performed at admission were repeated, and SIC and ISTH overt-DIC scores were calculated for each time point.

Simultaneously, fluid input and output during the preceding 24 h were recorded, allowing calculation of cumulative fluid balance (CFB) from the time of admission according to the following formula:

*CFB* = (*cumulative fluid input* − *cumulative fluid output*)/*body weight* × 100

CFB, expressed as a percentage of body weight, was used as a surrogate indicator of volemic status and was analyzed in relation to albumin levels. Fluid input was recorded as the total administered volume, whereas output included urine output measured by urinometer.

In addition, the presence of ongoing vasopressor therapy and continuous diuretic therapy was recorded at each reassessment. Therapeutic management, particularly fluid and vasopressor administration, was conducted according to the recommendations of the Surviving Sepsis Campaign. Since 2021, sepsis management in the IMCU has been regulated by an internal protocol based on these guidelines and applied uniformly. Prior to study initiation, departmental meetings were conducted to standardize clinical practices. During the study period, colloids were not used, in accordance with current guidelines, and any blood transfusions were administered according to clinical indication and appropriately documented.

#### 2.2.3. Outcomes

The primary outcomes of the study were the presence of sepsis-induced coagulopathy (SIC) and overt disseminated intravascular coagulation (ISTH-DIC). Both outcomes were analyzed as dichotomous variables (presence/absence), according to established criteria in the literature, as well as continuous scores. SIC was defined by an ISTH SIC score ≥ 4 [7], and overt DIC by an ISTH overt-DIC score ≥ 5 [8].

### 2.3. Statistical Analysis

Continuous variables were reported as mean (SD) or median (IQR), depending on their distribution. Categorical variables were expressed as counts and percentages. Univariate comparisons were performed using Fisher’s exact test, chi-square test, Student’s *t*-test, or Mann–Whitney U test, as appropriate.

The primary study outcomes (presence of SIC and ISTH overt DIC at follow-up time points) were modeled both as binary outcomes (presence/absence) and as continuous scores across the different analyses, depending on the specific model specification.

Multivariable longitudinal models based on generalized estimating equations (GEE) were used to assess the association of hemodynamic support therapies initiated in the early phase of sepsis in the IMCU setting. GEE models were specified with a correlation structure accounting for within-subject repeated measures, using a binomial distribution with a logit link for binary outcomes and a Gaussian distribution with an identity link for continuous outcomes. An exchangeable working correlation structure was specified. Robust (sandwich) standard errors were used for inference.

To ensure appropriate temporal ordering between exposure and outcome, variables related to therapies affecting perfusion were included as lagged covariates, referring to the previous time point. In addition, a lagged outcome variable was included in the models. defined as the presence of SIC/ISTH overt-DIC at the previous time point in binary models, and the previous SIC/ISTH overt-DIC score in continuous models. This approach explicitly accounted for the temporal dependence of coagulopathy and its intra-individual persistence over time. Because including a lagged outcome together with a non-independence working correlation can introduce dynamic-panel bias, the binary models were additionally re-estimated using an independence working correlation as a sensitivity analysis.

All multivariable models were adjusted for Charlson Comorbidity Index (CCI), admission SOFA score, PADUA score, IMPROVE score, IMPROVE bleeding score, and baseline anticoagulant therapy, categorized as prophylactic anticoagulation or chronic therapeutic anticoagulation. These variables were selected a priori based on their clinical plausibility as potential confounders in the relationship between hemodynamic strategies, anticoagulant therapy, and coagulopathy evolution. Highly collinear variables were not included simultaneously in the final models, and collinearity was assessed before model specification.

Sensitivity analyses were performed according to baseline SIC/ISTH overt-DIC status, distinguishing patients who were negative at baseline from those who were positive, in order to separately evaluate predictors of new-onset coagulopathy and those of persistence or resolution of pre-existing coagulopathy. For methodological consistency, the same covariates used in the global binary model were retained in subgroup analyses, with the exception of the lagged outcome variable, which was excluded to avoid over-adjustment within strata defined by baseline coagulopathy status.

Vasopressor exposure was selected a priori for the interaction analysis because vasoactive support, together with fluid administration, represents a central component of the initial hemodynamic management of sepsis and septic shock. A product term between vasopressor use and baseline SIC or overt DIC status was included to assess whether the association between vasoactive therapy and subsequent coagulopathy differed according to baseline coagulation status. Based on these models, adjusted predicted probabilities of SIC and overt DIC across levels of cumulative fluid balance were estimated, stratified by vasopressor exposure. Adjusted probabilities were computed as average marginal predictions over the observed covariate distribution, with the lagged outcome held at its observed values. Diuretic therapy was not selected for an interaction analysis because it is not routinely used as part of initial sepsis resuscitation, was administered to a smaller and clinically selected subgroup, and would therefore have provided limited power and potentially unstable interaction estimates. Nevertheless, diuretic exposure was included as a covariate in the multivariable models. Moreover, cumulative fluid balance incorporated both fluid input and output, including urine output, and therefore captured the overall net fluid balance, although not the specific pharmacological effect of diuretics.

Finally, a transition analysis between consecutive time points was performed to characterize the intra-individual temporal dynamics of coagulopathy. For this purpose, a four-level categorical variable was defined for each pair of consecutive observations, representing all possible transitions in SIC/ISTH overt-DIC status: persistent absence (SIC/ISTH overt-DIC − → SIC/ISTH overt-DIC −), new onset (− → +), resolution (+ → −), and persistence (+ → +). Transitions were analyzed descriptively using absolute and relative frequencies, both overall and stratified by initial time point. At each interval, the denominator was the total number of patients with evaluable observations at both consecutive time points. Therefore, the reported percentages describe the distribution of the four transition patterns within the entire evaluable cohort and do not represent conditional incidence or persistence probabilities calculated exclusively among patients at risk. Clinical and therapeutic characteristics associated with each transition pattern were compared using descriptive statistics. To examine factors associated with new-onset coagulopathy, a longitudinal GEE model was further constructed, restricted to observation pairs with negative baseline coagulopathy status, using transition to SIC/ISTH overt-DIC positivity (− → +) as the outcome. Given the limited number of incident events, the corresponding estimates are imprecise and are reported as exploratory.

All tests were two-sided, and a *p*-value < 0.05 was considered statistically significant. Given the number of models and outcomes examined, the analyses were considered exploratory and hypothesis-generating, and *p*-values were not adjusted for multiple comparisons. Analyses were performed using Stata version 16.2 (StataCorp, College Station, TX, USA).

## 3. Results

A total of 299 patients were included, corresponding to 1.447 longitudinal observations across the five predefined time points. The prevalence of SIC showed an initial increase from 36.8% (110/299) at admission to 47.5% (141/297) at 24 h, followed by a progressive decrease to 37.4% (108/289). 26.9% (76/283) and 20.8% (58/279) at 48, 72, and 96 h, respectively.

The prevalence of overt DIC rose marginally from 21.7% (65/299) at admission to 23.2% (69/297) at 24 h and then declined progressively to 18.0% (52/289) at 48 h, 14.8% (42/283) at 72 h, and 9.7% (27/279) at 96 h.

Among patients who did not undergo follow-up reassessments, the unavailability of reassessment data was solely due to early death (n = 20).

Baseline characteristics stratified by SIC and overt-DIC status at enrollment are summarized in Table 1. Patients who were SIC- or DIC-positive at admission presented with greater illness severity, reflected in higher SOFA and APACHE II scores, and a greater comorbidity burden (higher Charlson Comorbidity Index) than their negative counterparts. They also showed higher baseline thrombotic and bleeding risk, as indicated by the PADUA, IMPROVE, and IMPROVE bleeding scores, and a higher prevalence of active solid cancer and ongoing chemotherapy. Laboratory profiles differed accordingly, with higher creatinine, total bilirubin, and lactate, lower albumin and hemoglobin, lower platelet counts, prolonged INR and aPTT ratio, and lower fibrinogen among coagulopathy-positive patients; D-dimer was markedly higher in DIC-positive patients. Atrial fibrillation and prior anticoagulant therapy were more frequent among SIC-positive patients. Thirty-day mortality was higher in patients with coagulopathy at enrollment (SIC-positive 31.8% vs. 11.6%; DIC-positive 40.0% vs. 13.2%; both *p* < 0.001), whereas major bleeding and thrombotic events did not differ significantly by baseline status.

Comorbidity burden was characterized both using the CCI and by reporting individual conditions (Table 1).

In the global longitudinal analysis with binary outcomes, SIC dynamics were strongly driven by the presence of SIC at the previous reassessment, which represented the main determinant of subsequent positivity (OR 31.173, 95% CI 18.372–52.893; *p* < 0.001), with progressively decreasing odds at 48, 72, and 96 h. Cumulative fluid balance (CFB) and vasopressor use were not independently associated with subsequent SIC positivity, whereas diuretic therapy was associated with a significant increase in the probability of SIC positivity. A higher SOFA score at admission was associated with an increased likelihood of SIC during follow-up, while therapeutic anticoagulation was inversely associated with SIC positivity during follow-up (Table 2).

Similarly, overt-DIC dynamics were primarily driven by its persistence (OR 67.174, 95% CI 40.042–112.690; *p* < 0.001), with progressively decreasing odds at subsequent reassessments. Cumulative fluid balance, vasopressor use, and diuretic therapy were not independently associated with subsequent DIC positivity; only a higher admission SOFA score (OR 1.155, 95% CI 1.032–1.292; *p* = 0.012) and therapeutic anticoagulation (OR 0.286, 95% CI 0.138–0.590; *p* = 0.001) were inversely associated with subsequent overt DIC positivity (Table 3).

Stratified analyses according to baseline SIC and overt-DIC status showed different patterns across the two subgroups.

In initially negative patients, diuretic use was significantly associated with an increased risk of incident SIC (OR 2.200, 95% CI 1.225–3.951; *p* = 0.008), while the IMPROVE bleeding score was independently associated with the risk of incident SIC. No significant associations were observed with vasopressors, CCI, baseline SOFA score, or anticoagulation. For incident overt-DIC, CCI was independently associated with the risk of new onset, with a trend toward increased risk with norepinephrine use (OR 2.221, 95% CI 0.934–5.282; *p* = 0.071); therapeutic anticoagulation at enrollment was inversely associated with subsequent overt-DIC positivity among patients without overt DIC at baseline (OR 0.059, 95% CI 0.012–0.290; *p* < 0.001) (Table 4).

In initially positive patients, the probability of coagulopathy persistence decreased markedly over time for both outcomes, with progressively lower ORs at 48, 72, and 96 h. For SIC, the main factors associated with follow-up positivity were CCI, baseline SOFA score, and IMPROVE score, whereas therapeutic anticoagulation was inversely associated with SIC positivity during follow-up (OR 0.078, 95% CI 0.024–0.251; *p* < 0.001). For overt DIC, the main determinant of persistence was baseline SOFA score, with no significant associations observed for CFB, norepinephrine, or diuretic use (Table 4).

The interaction analysis did not identify significant effect modification of vasopressor use by baseline SIC status (OR 0.613, 95% CI 0.247–1.525; *p* = 0.293) or baseline overt-DIC status (OR 0.477, 95% CI 0.175–1.301; *p* = 0.148) (Table 5). Although the stratified models in Table 4 showed different point estimates across baseline-status subgroups, the interaction terms were not statistically significant.

In the SIC interaction model, coagulopathy dynamics were primarily driven by temporal persistence (OR 8.700, 95% CI 5.417–13.973; *p* < 0.001), with a progressive reduction in probability at subsequent reassessments. Diuretic use, baseline SOFA score, and IMPROVE bleeding score were associated with an increased probability of SIC positivity, whereas therapeutic anticoagulation was inversely associated with SIC positivity.

**Figure 1:**
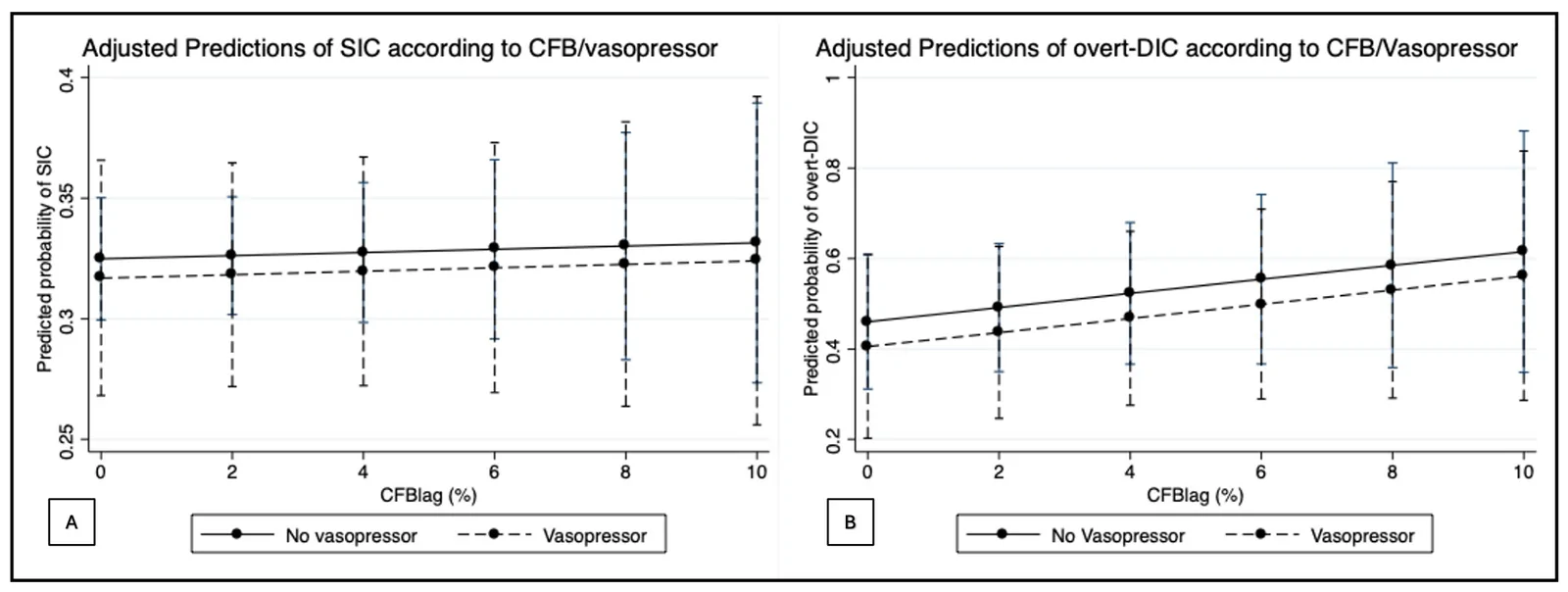
Adjusted predicted probabilities of SIC (**A**) and overt-DIC (**B**) according to cumulative fluid balance (CFB), stratified by vasoactive agent use at the preceding time point. Estimates were derived from the GEE model including the interaction term between vasopressor use and DIC. In both groups, the estimated probability of overt DIC progressively increased across the CFB gradient, although this trend was not statistically supported by the underlying model; the curves remained substantially parallel, consistent with the absence of a significant statistical interaction.

To further investigate the effect of early therapeutic interventions, in addition to the interaction analysis with norepinephrine use, adjusted predicted probabilities of SIC and overt-DIC were estimated according to cumulative fluid balance (CFB) at the preceding time point, stratified by exposure to vasoactive agents. For both outcomes, the curves were substantially parallel between patients exposed and not exposed to vasopressors. For SIC, the estimated probability increased modestly from 0.325 (95% CI 0.300–0.350) at neutral fluid balance to 0.332 (95% CI 0.274–0.389) at higher values, with a similar pattern among patients receiving vasopressors (from 0.317 to 0.324). For overt DIC, the gradient was more pronounced, increasing from 0.460 (95% CI 0.312–0.609) to 0.615 (95% CI 0.349–0.882) in patients not receiving vasopressors, and from 0.406 to 0.561 in those exposed to vasopressors. The parallelism of the curves, consistent with the absence of interaction in multivariable models, suggests that the association between volemic status and coagulopathy is independent of vasopressor use and may reflect an underlying pathophysiological mechanism; however, because the CFB coefficient did not reach statistical significance in any multivariable model and the confidence intervals around these predictions were wide and overlapping, this gradient should be interpreted as hypothesis-generating (Figure 1).

Across 1.148 consecutive transitions, the most frequent pattern was persistent absence of coagulopathy, observed in 58.1% (667/1148) of SIC transitions and 78.5% (901/1148) of DIC transitions, followed by persistence of coagulopathy in 27.9% (320/1148) and 13.8% (158/1148), respectively. Resolution occurred in 8.5% (98/1148) of SIC transitions and 5.0% (57/1148) of DIC transitions, whereas new onset was observed in 5.5% (63/1148) and 2.8% (32/1148), respectively.

Time-stratified analyses showed a progressive reduction in persistence for both outcomes. The proportion of persistent-positive transitions among all evaluable consecutive observation pairs decreased from 34.7% (103/297) to 18.6% (52/279) for SIC and from 18.5% (55/297) to 8.6% (24/279) for overt DIC. Similarly, new-onset transitions accounted for a progressively smaller proportion of all evaluable transitions, decreasing from 12.8% (38/297) to 2.2% (6/279) for SIC and from 4.7% (14/297) to 1.1% (3/279) for overt DIC. These percentages describe the distribution of transition patterns in the evaluable cohort rather than conditional incidence or persistence rates.

Transitions toward incident SIC were associated with higher CCI values (5.71 vs. 4.65; *p* = 0.004) and higher IMPROVE bleeding scores (7.75 vs. 6.71; *p* < 0.001), with a trend toward greater use of diuretics (*p* = 0.061). Incident overt DIC was associated with higher CCI values (6.94 vs. 5.03; *p* < 0.001), higher thrombotic and bleeding risk scores (PADUA: *p* = 0.041; IMPROVE: *p* = 0.028; IMPROVE bleeding: *p* = 0.006), and a lower frequency of baseline therapeutic anticoagulation (15.6% vs. 35.6%; *p* = 0.048).

In dedicated longitudinal models, the IMPROVE bleeding score emerged as an independent determinant of incident SIC (OR 1.191, 95% CI 1.028–1.380; *p* = 0.020), whereas incident DIC was independently associated with CCI (OR 1.323, 95% CI 1.132–1.545; *p* < 0.001), with trends observed for therapeutic anticoagulation (OR 0.301, 95% CI 0.087–1.042; *p* = 0.058) and vasoactive agent use (OR 2.988, 95% CI 0.768–11.631; *p* = 0.114).

For both outcomes, the probability of new onset was significantly lower at later time points, with a progressive reduction already evident from the first reassessment for SIC (OR 0.225, 95% CI 0.078–0.647; *p* = 0.006, decreasing to OR 0.077, 95% CI 0.020–0.302; *p* < 0.001 at the third reassessment) and from the third time point for DIC (OR 0.188, 95% CI 0.042–0.853; *p* = 0.030), indicating that coagulopathy incidence is predominantly concentrated in the early phases of sepsis.

Sensitivity analyses supported the robustness of the primary models. Re-estimating the global binary GEE models using an independence working correlation structure yielded effect estimates that were highly consistent with those obtained in the primary exchangeable-correlation analysis (Supplementary Table S1). For SIC, the strongest predictor of subsequent coagulopathy remained SIC positivity at the previous assessment (OR 28.93, 95% CI 17.13–48.86; *p* < 0.001), while admission SOFA score remained independently associated with SIC occurrence during follow-up (OR 1.25, 95% CI 1.13–1.38; *p* < 0.001). Therapeutic anticoagulation remained inversely associated with SIC positivity (OR 0.51, 95% CI 0.31–0.83; *p* = 0.007), and diuretic therapy remained associated with an increased probability of SIC positivity (OR 2.15, 95% CI 1.32–3.51; *p* = 0.002).

Similarly, for overt-DIC, the presence of overt-DIC at the previous assessment remained the dominant determinant of subsequent positivity (OR 49.07, 95% CI 26.76–89.97; *p* < 0.001). Admission SOFA score was independently associated with overt DIC during follow-up (OR 1.16, 95% CI 1.03–1.30; *p* = 0.012), whereas therapeutic anticoagulation remained associated with a significantly lower probability of overt DIC (OR 0.43, 95% CI 0.23–0.80; *p* = 0.008). No relevant differences in the direction, magnitude, or statistical significance of the main associations were observed compared with the primary exchangeable-correlation models, supporting the robustness of the findings to the choice of working correlation structure.

## 4. Discussion

This longitudinal repeated-measures study in critically ill patients with sepsis demonstrated that SIC and ISTH overt-DIC are not merely dynamic conditions, but rather processes strongly constrained by an early temporal dependence. Indeed, the coagulation status observed at subsequent time points was largely determined by the early development of sepsis-related coagulopathy, resulting in a marked autoregressive and self-perpetuating pattern. These findings suggest that the trajectory of coagulopathy in sepsis is established early and subsequently tends to stabilize over time. Our results are consistent with the pathophysiological model proposed by Levi and colleagues, according to which coagulation activation in sepsis represents a self-amplifying process, closely intertwined with the inflammatory response and progressively less reversible as the disease evolves [3,11,12].

Our data further support the hypothesis that coagulopathy is an early event in the course of sepsis. The incidence of SIC and DIC was highest within the first 24–48 h, with a progressive reduction in new diagnoses at later time points. This pattern suggests that coagulation activation occurs during the initial phases of the disease, in parallel with the cytokine storm and endothelial dysfunction [13,14]. These observations are consistent with the concept of an early pathophysiological “hit” capable of determining the coagulative trajectory at an early stage, as proposed in immunothrombosis models [5]. In this context, the endothelium appears to play a central role, and among its functions, regulation of the balance between procoagulant and anticoagulant activity seems particularly vulnerable to inflammatory stimuli, representing one of the earliest systems to become impaired. This alteration, which is highly dynamic, may develop rapidly but may also resolve early following control of the injurious stimulus, unlike other endothelial functions, such as regulation of vascular permeability, which tend to persist longer over time [4,15,16].

Within this framework, SIC and DIC appear to represent distinct stages along a pathophysiological continuum of sepsis-associated coagulopathy. SIC appears to be earlier, more frequent, and more dynamic, consistent with an initial and potentially reversible phase; conversely, DIC represents an overt form of coagulation dysfunction, reflecting a more advanced and likely less reversible stage [17,18].

Regarding factors associated with coagulopathy. Sepsis severity was reflected by the SOFA score and was independently associated with both the presence and severity of coagulation abnormalities. Higher SOFA scores, indicative of more extensive organ dysfunction, may reflect a greater degree of systemic inflammatory activation and, at the same time, indirectly contribute to coagulopathy through mechanisms related to tissue dysfunction. In this context, organ damage may further sustain and amplify endothelial dysfunction, thereby promoting persistence of coagulation abnormalities [19,20].

Therapeutic-dose anticoagulation at enrollment was consistently associated with lower subsequent SIC and overt-DIC positivity across several analyses. However, this inverse association cannot be interpreted as evidence of a treatment effect. Anticoagulation was prescribed for independent clinical indications rather than specifically for sepsis-associated coagulopathy, and treated patients may have differed from untreated patients in several measured and unmeasured characteristics, including atrial fibrillation, cardiovascular and thrombotic risk, malignancy, bleeding risk, and clinical severity. In addition, therapeutic anticoagulation may have been withheld from patients with thrombocytopenia, active bleeding, severe coagulation abnormalities, or a high perceived bleeding risk, resulting in treatment-selection or contraindication bias.

Previous studies have evaluated anticoagulant strategies in selected populations with sepsis, with conflicting results from randomized trials of antithrombin, heparin, and recombinant thrombomodulin [21,22,23,24,25,26]. Although our findings identify an inverse association worthy of further investigation, they do not demonstrate that therapeutic anticoagulation prevents coagulopathy, promotes its resolution, or identifies a therapeutic window. Dedicated prospective interventional studies are required to determine whether anticoagulation can modify the trajectory of sepsis-associated coagulopathy and which patients, if any, might benefit.

Beyond treatment timing and conventional clinical risk factors, the biological heterogeneity of sepsis may influence the observed response to anticoagulant therapy. Seymour et al. identified four clinical sepsis phenotypes (α, β, γ, and δ), with the δ phenotype showing the highest mortality and more severe organ dysfunction and coagulation activation [27]. As reviewed by Yu et al., this phenotype was also characterized by increased thrombin–antithrombin complexes, D-dimer, and plasminogen activator inhibitor-1 [28]. In our study, D-dimer was assessed longitudinally as part of the coagulation profile, whereas thrombin–antithrombin complexes and plasminogen activator inhibitor-1 were not measured [28]. Future interventional studies should evaluate whether combining these biomarkers with dynamic SIC and overt-DIC assessment can identify patients with marked coagulation activation for enrollment in trials of anticoagulant strategies. However, these biomarkers are not yet validated for selecting patients for anticoagulation in routine clinical practice.

The role of hemodynamic and perfusion management in coagulopathy appears more complex and likely requires further investigation. The association between cumulative fluid balance (CFB) and DIC, independent of vasopressor use, suggests a potential link between fluid overload, endothelial dysfunction, and activation of coagulation pathways. This is consistent with evidence on endothelial glycocalyx damage and microcirculatory alterations in sepsis [4,29,30].

Fluid overload and the tissue effects of hyperhydration may disrupt several key mechanisms of homeostasis, including those regulating the balance between procoagulant and anticoagulant systems, which are strongly dependent on endothelial integrity [31]. However, the overall impact of hemodynamic alterations and perfusion-related mechanisms appears secondary to the inflammatory component, which directly affects the endothelium, whereas hemodynamic factors exert a predominantly indirect effect through the interaction between the vascular compartment and tissues. It is plausible that a more refined modeling of these phenomena, together with the study of more homogeneous cohorts in terms of disease phase, could better clarify the role of hemodynamics, and therefore of the fluid–vasopressor balance. in determining the evolution of sepsis-associated coagulopathy.

Finally, the transition analyses provide additional insight into the natural history of sepsis-associated coagulopathy. The maintenance of a non-coagulopathic state represents the most frequent trajectory, followed by persistence of coagulopathy, whereas new onset is relatively rare and predominantly confined to the early phases of the disease. This pattern suggests that patients who do not develop coagulopathy in the early stages have a reduced likelihood of developing it later on [7,8,9]. The association between incident coagulopathy, comorbidity burden, and bleeding risk underscores the role of individual susceptibility in shaping the evolution of the coagulation profile.

Taken together, these findings reinforce the importance of early identification and dynamic monitoring of septic patients, as coagulopathy, similarly to perfusion abnormalities, represents an evolving process that is largely established in the early phases of the disease. This highlights the need to integrate patient-specific characteristics into prognostic stratification models, in order to identify individuals at higher risk of progression and to consider targeted interventions aimed at modulating coagulation during the early. potentially more reversible phase of the disease trajectory.

The study has several limitations.

First, its single-center design exposes it to biases inherent to this setting and potentially related to local routine clinical practices. However, the strong standardization of both assessment and management, based on established guidelines, should support the generalizability of the findings.

Second, the observational design precludes causal inference regarding the relationship between therapeutic anticoagulation and the subsequent coagulation trajectory. Anticoagulation was not randomly assigned but was prescribed for clinical indications unrelated to the treatment of sepsis-associated coagulopathy. Treated and untreated patients may therefore have differed in atrial fibrillation, cardiovascular and thrombotic risk, malignancy, bleeding risk, illness severity, and other measured or unmeasured characteristics. Conversely, therapeutic anticoagulation may have been withheld from patients with thrombocytopenia, active bleeding, more severe coagulation abnormalities, or a high perceived bleeding risk, introducing treatment-selection or contraindication bias. Although the analyses were adjusted for several indicators of comorbidity, disease severity, thrombotic risk, and bleeding risk, residual confounding remains possible. Therefore, the observed inverse association should not be interpreted as evidence that therapeutic anticoagulation prevents or accelerates the resolution of SIC or overt DIC.

Third, therapies affecting hemodynamics, such as diuretics, vasopressors, and fluid management strategies, should be interpreted as proxies of underlying clinical conditions rather than direct causal determinants. In particular, cumulative fluid balance represents an indirect measure of volemic status and may not accurately reflect fluid distribution or microcirculatory alterations. However, the consistency of the findings across multivariable analyses and predicted probability models, as well as the absence of interaction with vasopressor use, strengthens the hypothesis of an independent and physiopathologically plausible association.

Fourth, specific biomarkers of endothelial activation, inflammation, or fibrinolysis were not systematically assessed, which could have provided further insight into the underlying pathophysiological mechanisms. Nevertheless, the primary aim of the study was clinical and epidemiological in nature, and the use of routinely available parameters enhances the external validity and clinical applicability of the findings.

Finally, the follow-up limited to the first 96 h does not allow for evaluation of the medium- to long-term evolution of coagulopathy or its association with late clinical outcomes. However, focusing on the early phase of sepsis enables a high-resolution temporal characterization of the period in which the coagulation trajectory is established, which is likely the most physiologically relevant window and the one with the greatest potential therapeutic impact.

## 5. Conclusions

Sepsis-associated coagulopathy is an early, dynamic process that is strongly shaped by its initial trajectory. SIC and DIC appear to represent progressive stages along a pathophysiological continuum, in which coagulation activation tends to become established in the early phases of the disease and persist over time. In this context, clinical severity and individual susceptibility were associated with the evolution of the coagulation profile, whereas associations with therapeutic strategies require cautious interpretation.

The inverse association between therapeutic anticoagulation and subsequent coagulopathy is hypothesis-generating and may reflect baseline differences and clinical treatment selection. It does not establish a protective or disease-modifying effect.

Overall, these findings reinforce the need for early and longitudinal monitoring of coagulation in septic patients and support a more selective, time-sensitive, and phenotype-driven approach aimed at identifying patients in whom modulation of coagulation may translate into clinical benefit.

## Figures and Tables

**Table 1:** Baseline characteristics of the study population (n = 299), stratified by SIC and ISTH overt-DIC status at enrollment.

	SIC Status			ISTH Overt-DIC Status		
Variable	SIC Negative (n = 189)	SIC Positive (n = 110)	*p*-Value	ISTH-DIC Negative (n = 234)	ISTH-DIC Positive (n = 65)	*p*-Value
Demographics						
Age. years	72.0 [62.0–80.0]	75.5 [67.2–82.8]	0.009	74.0 [63.0–80.0]	75.0 [65.0–83.0]	0.383
Male sex	113 (59.8)	74 (67.3)	0.244	138 (59.0)	49 (75.4)	0.023
Body mass index. kg/m^2^	26.2 [23.6–29.3]	24.4 [22.0–27.4]	0.014	26.1 [23.7–29.3]	23.7 [22.1–26.6]	0.008
Severity scores						
SOFA score	3.0 [3.0–5.0]	5.0 [4.0–7.0]	<0.001	4.0 [3.0–5.0]	6.0 [4.0–8.0]	<0.001
APACHE II score	12.0 [9.0–15.0]	14.0 [11.0–17.8]	<0.001	12.0 [9.2–15.0]	15.0 [11.0–18.0]	0.005
NEWS at admission	6.0 [3.0–8.0]	5.0 [3.2–8.0]	0.547	6.0 [3.0–8.0]	5.0 [3.0–7.0]	0.440
Charlson Comorbidity Index	4.0 [3.0–7.0]	6.0 [4.0–8.0]	<0.001	5.0 [3.0–7.0]	6.0 [4.0–8.0]	0.025
Thrombosis and bleeding risk						
PADUA score	3.0 [2.0–4.0]	4.0 [2.0–6.0]	<0.001	3.0 [2.0–4.0]	4.0 [2.0–5.0]	0.040
IMPROVE VTE score	2.5 [2.5–2.5]	2.5 [2.5–4.5]	<0.001	2.5 [2.5–2.5]	2.5 [2.5–4.5]	<0.001
IMPROVE bleeding score	6.0 [5.0–7.5]	9.5 [7.5–11.0]	<0.001	6.5 [5.0–8.5]	9.5 [7.5–11.1]	<0.001
Comorbidities						
Hypertension	121 (64.0)	65 (59.1)	0.469	151 (64.5)	35 (53.8)	0.154
Diabetes mellitus	47 (24.9)	31 (28.2)	0.622	62 (26.5)	16 (24.6)	0.884
Chronic ischemic heart disease	25 (13.2)	13 (11.8)	0.863	32 (13.7)	6 (9.2)	0.459
Atrial fibrillation	34 (18.0)	48 (43.6)	<0.001	65 (27.8)	17 (26.2)	0.918
Chronic heart failure	24 (12.7)	19 (17.3)	0.360	35 (15.0)	8 (12.3)	0.735
COPD	27 (14.3)	12 (10.9)	0.511	36 (15.4)	3 (4.6)	0.022
Chronic kidney disease	31 (16.4)	26 (23.6)	0.167	44 (18.8)	13 (20.0)	0.969
Chronic liver disease	2 (1.1)	6 (5.5)	0.055	4 (1.7)	4 (6.2)	0.071
Previous stroke	12 (6.3)	10 (9.1)	0.518	18 (7.7)	4 (6.2)	0.794
Active solid cancer	26 (13.8)	40 (36.4)	<0.001	41 (17.5)	25 (38.5)	<0.001
Active chemotherapy	12 (6.3)	25 (22.7)	<0.001	22 (9.4)	15 (23.1)	0.006
Dementia	6 (3.2)	10 (9.1)	0.054	15 (6.4)	1 (1.5)	0.209
Bedridden	8 (4.2)	6 (5.5)	0.843	12 (5.1)	2 (3.1)	0.742
Prior therapy						
Previous anticoagulant therapy	25 (13.2)	50 (45.5)	<0.001	56 (23.9)	19 (29.2)	0.478
Previous antiplatelet therapy	37 (19.6)	15 (13.6)	0.251	43 (18.4)	9 (13.8)	0.504
Laboratory at admission						
Creatinine. mg/dL	1.3 [1.0–2.0]	1.7 [1.2–2.5]	<0.001	1.4 [1.0–2.2]	1.7 [1.2–3.1]	0.010
Total bilirubin. mg/dL	0.9 [0.5–1.5]	1.3 [0.8–2.1]	<0.001	0.9 [0.5–1.6]	1.3 [0.8–2.0]	0.004
Albumin. g/dL	2.6 [2.2–3.0]	2.4 [2.1–2.8]	0.002	2.6 [2.2–3.0]	2.3 [2.0–2.8]	0.002
C-reactive protein. mg/L	15.3 [9.0–23.6]	15.4 [7.5–22.6]	0.483	15.2 [8.7–23.0]	16.0 [8.0–22.8]	0.787
Lactate. mmol/L	1.5 [1.1–2.5]	2.0 [1.4–3.3]	<0.001	1.5 [1.1–2.5]	2.6 [1.7–3.7]	<0.001
Hemoglobin. g/dL	12.3 [10.2–14.0]	11.3 [9.6–12.8]	0.001	12.2 [10.2–13.9]	11.3 [8.5–12.8]	0.002
Platelet count. × 10^9^/L	192.5 [143.2–269.5]	100.5 [62.8–147.0]	<0.001	177.0 [129.2–258.0]	83.0 [58.0–124.2]	<0.001
White blood cell count. × 10^9^/L	12.9 [8.6–18.4]	10.1 [4.0–17.1]	0.003	12.9 [8.2–18.4]	8.5 [2.9–14.3]	<0.001
INR	1.2 [1.1–1.3]	1.5 [1.3–1.9]	<0.001	1.2 [1.1–1.4]	1.4 [1.3–1.8]	<0.001
aPTT ratio	1.2 [1.0–1.3]	1.4 [1.2–1.7]	<0.001	1.2 [1.0–1.4]	1.4 [1.2–1.6]	<0.001
Fibrinogen. mg/dL	571.0 [433.0–810.0]	466.5 [381.8–611.8]	<0.001	561.0 [430.0–788.8]	454.0 [302.0–562.0]	<0.001
D-dimer. ng/mL	2227.0 [1432.0–4470.0]	2871.0 [1069.8–5997.0]	0.536	1910.0 [1009.2–3980.0]	7325.1 [3336.0–14,389.0]	<0.001
Anticoagulation at enrollment						
Prophylactic anticoagulation	101 (53.4)	26 (23.6)	<0.001	108 (46.2)	19 (29.2)	0.023
Therapeutic anticoagulation	47 (24.9)	46 (41.8)	0.003	79 (33.8)	14 (21.5)	0.088
Coagulopathy scores						
SIC score	2.0 [1.0–3.0]	4.0 [4.0–5.0]	<0.001	3.0 [2.0–3.0]	4.0 [4.0–5.0]	<0.001
ISTH overt-DIC score	2.0 [1.0–3.0]	4.0 [3.0–6.0]	<0.001	2.0 [1.0–3.0]	6.0 [5.0–6.0]	<0.001
Outcomes						
30-day mortality	22 (11.6)	35 (31.8)	<0.001	31 (13.2)	26 (40.0)	<0.001
Major bleeding	4 (2.1)	2 (1.8)	1.000	4 (1.7)	2 (3.1)	0.614
Thrombotic event	12 (6.3)	10 (9.1)	0.518	14 (6.0)	8 (12.3)	0.144

**Table 2:** Global longitudinal models for SIC: binary and continuous outcomes. Multivariable GEE models with an exchangeable correlation structure. The binary model included SIC_lag; the continuous model included the previous SIC score.

Variable	SIC (Binary) OR (95% CI)	*p*-Value	SIC (Continuous) β (95% CI)	*p*-Value
Reassessment 2	0.138 (0.070–0.273)	<0.001	−0.860 (−1.019–−0.701)	<0.001
Reassessment 3	0.090 (0.045–0.178)	<0.001	−1.105 (−1.269–−0.941)	<0.001
Reassessment 4	0.089 (0.045–0.173)	<0.001	−0.961 (−1.112–−0.810)	<0.001
CFB	1.005 (0.943–1.071)	0.875	0.015 (−0.001–0.031)	0.072
Vasopressor use	0.894 (0.547–1.461)	0.654	0.043 (−0.080–0.165)	0.494
Diuretic use	2.161 (1.319–3.540)	0.002	0.209 (0.096–0.323)	<0.001
SIC/SIC_score	31.173 (18.372–52.893)	<0.001	0.752 (0.698–0.807)	<0.001
CCI	1.054 (0.969–1.145)	0.219	0.021 (−0.001–0.042)	0.056
Baseline SOFA score	1.246 (1.129–1.376)	<0.001	0.041 (0.013–0.068)	0.003
PADUA score	0.907 (0.801–1.026)	0.121	−0.015 (−0.044–0.014)	0.307
IMPROVE score	1.004 (0.827–1.219)	0.968	−0.015 (−0.060–0.029)	0.498
IMPROVE bleeding score	1.211 (1.092–1.342)	<0.001	0.041 (0.016–0.067)	0.001
Prophylactic anticoagulation at enrollment	0.684 (0.423–1.106)	0.121	−0.061 (−0.188–0.065)	0.341
Therapeutic anticoagulation at enrollment	0.509 (0.310–0.833)	0.007	−0.115 (−0.237–0.008)	0.066

**Table 3:** Global longitudinal models: binary and continuous outcomes. Multivariable GEE models with an exchangeable correlation structure. The binary model included DIC_lag; the continuous model included ISTH_DIC_lag.

Variable	Overt-DIC (Binary) OR (IC 95%)	*p*-Value	ISTH-DIC (Continuous) β (IC 95%)	*p*-Value
Reassessment 2	0.358 (0.153–0.836)	0.018	−0.395 (−0.589–−0.201)	<0.001
Reassessment 3	0.415 (0.173–0.999)	0.050	−0.448 (−0.646–−0.251)	<0.001
Reassessment 4	0.253 (0.106–0.602)	0.002	−0.683 (−0.871–−0.495)	<0.001
CFB	0.998 (0.919–1.083)	0.955	0.015 (−0.005–0.036)	0.149
Vasopressor use	1.516 (0.833–2.758)	0.173	0.033 (−0.109–0.175)	0.653
Diuretic use	0.944 (0.523–1.705)	0.849	0.107 (−0.029–0.243)	0.123
DIC/ISTH_DIC	67.174 (40.042–112.690)	<0.001	0.814 (0.779–0.848)	<0.001
CCI	1.051 (0.943–1.172)	0.371	0.003 (−0.021–0.028)	0.781
Baseline SOFA score	1.155 (1.032–1.292)	0.012	0.038 (0.010–0.066)	0.008
PADUA score	0.971 (0.824–1.144)	0.721	−0.029 (−0.065–0.008)	0.121
IMPROVE score	1.091 (0.829–1.435)	0.535	0.026 (−0.037–0.089)	0.414
Prophylactic anticoagulation at initiation	0.671 (0.381–1.181)	0.167	−0.134 (−0.269–−0.002)	0.053
Therapeutic anticoagulation at initiation	0.286 (0.138–0.590)	0.001	−0.214 (−0.356–−0.073)	0.003

**Table 4:** (**A**) Risk prediction model for SIC among patients without SIC at baseline. (**B**) Risk prediction model for over-DIC among patients without over-DIC at baseline.

**A**	**Negative SIC at Baseline**		**Negative OVER-DIC at Baseline**	
**Variable**	**OR (IC95%)**	***p*** **-Value**	**OR (IC95%)**	***p*** **-Value**
Reassessment 2	0.485 (0.262–0.896)	0.021	0.614 (0.217–1.740)	0.359
Reassessment 3	0.244 (0.126–0.473)	<0.001	0.582 (0.204–1.663)	0.312
Reassessment 4	0.116 (0.049–0.278)	<0.001	0.362 (0.123–1.064)	0.065
CFB	0.997 (0.880–1.130)	0.960	0.958 (0.856–1257)	0.475
Vasopressor use	1.159 (0.530–2.535)	0.711	2.221 (0.934–5.282)	0.071
Diuretic use	2.200 (1.225–3.951)	0.008	1.085 (0.469–2.506)	0.849
CCI	0.992 (0.858–1.147)	0.916	1.265 (1.072–1.494)	0.005
Baseline SOFA score	0.912 (0.754–1.104)	0.345	1.002 (0.826–1.216)	0.981
PADUA score	0.808 (0.635–1.029)	0.083	0.812 (0.634–1.039)	0.098
IMPROVE score	1.182 (0.758–1.843)	0.461	1.255 (0.835–1.885)	0.275
IMPROVE bleeding score	1.375 (1.134–1.681)	0.001	1.176 (1.014–1.364)	0.032
Prophylactic anticoagulation at initiation	0.898 (0.360–2.241)	0.817	0.797 (0.333–1.911)	0.611
Therapeutic anticoagulation at initiation	1.013 (0.406–2.525)	0.979	0.059 (0.012–0.290)	<0.001
**B**	**Positive SIC at Baseline**		**Positive OVER-DIC at Baseline**	
**Variable**	**OR (IC95%)**	***p*** **-Value**	**OR (IC95%)**	***p*** **-Value**
Reassessment 2	0.106 (0.034–0.327)	<0.001	0.217 (0.075–0.629)	0.005
Reassessment 3	0.046 (0.014–0.159)	<0.001	0.125 (0.041–0.380)	<0.001
Reassessment 4	0.032 (0.009–0.108)	<0.001	0.056 (0.019–0.166)	<0.001
CFB	1.000 (0.928–1.076)	0.992	1.065 (0.947–1.197)	0.293
Vasopressor use	0.776 (0.395–1.522)	0.460	0.800 (0.359–1.784)	0.586
Diuretic use	1.519 (0.729–3.163)	0.265	1.396 (0.609–3.201)	0.431
CCI	1.365 (1.124–1.657)	0.002	0.861 (0.705–1.051)	0.142
Baseline SOFA score	1.482 (1.181–1.860)	0.001	1.325 (1.100–1.596)	0.003
PADUA score	0.900 (0.741–1.093)	0.288	1.296 (0.937–1.793)	0.118
IMPROVE score	0.781 (0.627–0.973)	0.027	1.138 (0.692–1.871)	0.611
IMPROVE bleeding score	0.946 (0.790–1.132)	0.546	1.095 (0.901–1.331)	0.361
Prophylactic anticoagulation at initiation	0.311 (0.087–1.105)	0.071	0.501 (0.191–1.313)	0.160
Therapeutic anticoagulation at initiation	0.078 (0.024–0.251)	<0.001	0.709 (0.256–1.966)	0.509

**Table 5:** Interaction analysis between vasopressor use and baseline SIC/overt-DIC status.

	Interaction Analysis Between Vasopressor Use and Baseline SIC Status		Interaction Analysis Between Vasopressor Use and Baseline Overt-DIC Status	
Variable	OR (IC 95%)	*p*-Value	OR (IC 95%)	*p*-Value
Reassessment 2	0.178 (0.097–0.327)	<0.001	0.435 (0.204–0.927)	0.031
Reassessment 3	0.101 (0.056–0.184)	<0.001	0.382 (0.176–0.823)	0.015
Reassessment 4	0.080 (0.044–0.147)	<0.001	0.195 (0.089–0.426)	<0.001
CFB_lag	1.007 (0.947–1.070)	0.830	0.994 (0.919–1.075)	0.879
Vasopressor_lag	1.149 (0.559–2.363)	0.706	1.167 (0.667–4.500)	0.109
SIC_baseline	4.443 (2.425–8.141)	<0.001	9.463 (4.147–21.596)	<0.001
Vasopressor_lag x SIC_baseline	0.613 (0.247–1.525)	0.293	0.477 (0.175–1.301)	0.148
DIURETIC_lag	2.118 (1.315–3.414)	0.002	0.979 (0.538–1.782)	0.945
SIC_POSITIVE_lag	8.700 (5.417–13.973)	<0.001	7.887 (4.219–14.742)	<0.001
CCI	1.054 (0.964–1.153)	0.248	1.133 (1.030–1.246)	0.010
Baseline SOFA score	1.173 (1.053–1.307)	0.004	1.180 (1.050–1.327)	0.006
PADUA score	0.874 (0.765–0.998)	0.048	0.990 (0.842–1.162)	0.898
IMPROVE score	1.007 (0.804–1.260)	0.954	1.046 (0.815–1.343)	0.723
IMPROVE bleeding score	1.227 (1.093–1.377)	0.001	1.130 (0.980–1.303)	0.092
Prophylactic anticoagulation at initiation	0.648 (0.374–1.123)	0.122	0.734 (0.362–1.486)	0.390
Therapeutic anticoagulation at initiation	0.410 (0.235–0.715)	0.002	0.370 (0.183–0.745)	0.005

## Data Availability

Data are unavailable due to privacy restrictions.

## Supplementary Materials

The following supporting information can be downloaded at: https://www.mdpi.com/article/10.3390/jcm15166285/s1, Table S1: Sensitivity analysis of the global binary GEE models. Effect estimates obtained using an independence working correlation structure were highly consistent with those from the primary analysis assuming an exchangeable working correlation structure, supporting the robustness of the main findings.

## Abbreviations

The following abbreviations are used in this manuscript:

SIC	sepsis-induced coagulopathy
DIC	disseminated intravascular coagulation
GEE	generalized estimating equation
ISTH	International Society on Thrombosis and Haemostasias
IMCU	Intermediate Care Unit
ED	Emergency Department
SOFA	Sequential Organ Failure Assessment
CCI	Charlson Comorbidity Index
NEWS	National Early Warning Score
CFB	cumulative fluid balance
