# Supplementary material for: Early Evolution of Sepsis-Associated Coagulopathy and Its Association with Therapeutic Exposures: A Prospective Longitudinal Cohort Study
Source: J Clin Med. 2026 Aug 13;15(16):6285. doi: 10.3390/jcm15166285 (PMC13513265; doi:10.3390/jcm15166285)
Supplement: Supplementary file 1 [file jcm-15-06285-s001.zip › jcm-4443223-supplementary.pdf]

Journal of Clinical Medicine

MDPI

Supplementary Table S1. Sensitivity analysis of the global binary GEE models. Effect estimates obtained using an independence working correlation structure were highly consistent with those from the primary analysis assuming an exchangeable working correlation structure, supporting the robustness of the main findings.

	SIC		DIC	
**Variable**	**Primary (exchangeable) OR (95% CI)**	**Independence OR (95% CI)**	**Primary (exchangeable) OR (95% CI)**	**Independence OR (95% CI)**
Reassessment 2	0.138 (0.070–0.273)	0.142 (0.073–0.277)	0.363 (0.126–1.040)	0.383 (0.147–1.002)
Reassessment 3	0.090 (0.045–0.178)	0.092 (0.047–0.179)	0.411 (0.163–1.035)	0.413 (0.177–0.960)
Reassessment 4	0.089 (0.045–0.173)	0.089 (0.046–0.172)	0.208 (0.084–0.514)	0.205 (0.088–0.477)
CFB_lag	1.005 (0.943–1.071)	1.005 (0.943–1.071)	0.984 (0.908–1.066)	0.992 (0.927–1.061)
Vasopressor_lag	0.894 (0.547–1.461)	0.894 (0.548–1.458)	1.281 (0.678–2.419)	1.244 (0.677–2.287)
Diuretic_lag	2.161 (1.319–3.540)	2.151 (1.317–3.513)	1.101 (0.530–2.287)	1.057 (0.528–2.117)
SIC_lag / DIC_lag	31.173 (18.372–52.893)	28.927 (17.126–48.861)	68.255 (37.108–125.546)	49.069 (26.762–89.971)
CCI	1.054 (0.969–1.145)	1.052 (0.968–1.144)	1.052 (0.942–1.174)	1.040 (0.936–1.155)
Baseline SOFA score	1.246 (1.129–1.376)	1.253 (1.135–1.383)	1.132 (1.005–1.276)	1.159 (1.033–1.299)
PADUA score	0.907 (0.801–1.026)	0.906 (0.801–1.025)	0.971 (0.837–1.126)	0.978 (0.845–1.132)
IMPROVE score	1.004 (0.827–1.219)	1.005 (0.829–1.218)	1.087 (0.871–1.357)	1.088 (0.873–1.355)
IMPROVE bleeding score	1.211 (1.092–1.342)	1.216 (1.096–1.349)	1.113 (0.982–1.261)	1.124 (0.997–1.267)
Prophylactic anticoagulation	0.684 (0.423–1.106)	0.679 (0.419–1.098)	0.733 (0.391–1.374)	0.702 (0.386–1.277)
Therapeutic anticoagulation	0.509 (0.310–0.833)	0.507 (0.310–0.830)	0.456 (0.238–0.875)	0.427 (0.227–0.804)
